# The dynamics of microbial community structure and metabolic function in different parts of cigar tobacco leaves during air-curing

**DOI:** 10.3389/fmicb.2024.1438566

**Published:** 2024-12-12

**Authors:** Wenlong Li, Jun Yu, Hao Li, Chunlei Yang, Zheng Peng, Juan Zhang

**Affiliations:** ^1^Key Laboratory of Industrial Biotechnology, Ministry of Education, School of Biotechnology, Jiangnan University, Wuxi, China; ^2^Science Center for Future Foods, Jiangnan University, Wuxi, China; ^3^Hubei Branch of China National Tobacco Corporation, Wuhan, China; ^4^Hubei Tobacco Research Institute, Wuhan, China

**Keywords:** cigar tobacco leaves, microbial community, metabolic function, air-curing, HS-SPME-GC–MS

## Abstract

Air-curing is the initial step in the processing of cigar tobacco leaves. However, the dynamics of microbial community and metabolic functions in different parts of tobacco leaves during this process remain largely unclear. In this study, amplicon-based high-throughput sequencing revealed that *Pseudomonas* (9.0 to 29.9%) and *Sphingomonas* (0.5 to 13.8%) were the dominant bacterial genera in the early stages of air-curing, while *Pantoea* (1.7–90.4%) became predominant after air-curing. Microbial community diversity analysis indicated that species richness and diversity were significantly higher during the fresh leaf and withering periods. Functional prediction based on PICRUSt2 suggested that the microbial communities in the middle leaves exhibited higher abundances of metabolic pathways related to carbohydrates and amino acids than those in the upper leaves, potentially leading to the formation of more flavor compounds. The volatile flavor compounds were detected during the air-curing process by HS-SPME-GC–MS, with alkaloids and esters being the most prominent, although their accumulation periods differed across leaf parts. Furthermore, based on PLS-DA, 17 and 38 significantly changed flavor components were identified in the upper and middle leaves, respectively. Finally, the potential relationships between characteristic microbes and flavor components were explored based on *Spearman* correlation coefficient. It was found that multiple bacteria such as *Rhodanobacter*, *Gemmatimonas*, and *Ramlibacter* present in the middle leaves exhibited significant positive correlations with multiple flavor compounds such as 3,3-dimethylacrylic acid, phenylacetone, 2,3-butanedione, and geranylacetone, potentially promoting the flavor formation of cigar tobacco leaves during air-curing process. This study provides scientific insights into the role of microorganisms during the air-curing process of cigar tobacco leaves and offers a scientific basis for screening of specific functional microorganisms to improve and stabilize cigar tobacco flavor in the future.

## 1 Introduction

Cigars are globally renowned premium tobacco products known for their rich aroma, balanced bitterness, and captivating qualities (Zhang et al., 2024). The production process of cigars is intricate, involving various stages such as cultivation, air-curing, agricultural fermentation, industrial fermentation, rolling, and aging of tobacco leaves (Zhang et al., 2023b). Among these, air-curing is the initial step in processing, where harvested tobacco leaves are dried in well-ventilated curing barns with controlled temperature and humidity. During this process, influenced by environmental factors like temperature and humidity, and microbial metabolic activities, the moisture content in the leaves decreases gradually, leading to internal transformations and a darkening hue from yellow to brown (Xing et al., 2024).

The air-curing and fermentation of cigar tobacco leaves typically occur in an open environment where spontaneous microbial colonization and metabolic activities generate a large number of flavor compounds, thereby impacting the quality and aroma of cigar tobacco (Zhang et al., 2023b). Previous studies have shown the presence of a diverse microbial community in cigar tobacco leaves. A recent survey identified 89 discrete bacterial genera and 19 fungal genera in commercial tobacco products, identifying *Bacillus*, *Pseudomonas*, and *Staphylococcus* as the dominant bacteria, while *Aspergillus* and *Penicillium* were common in fungal communities (Chattopadhyay et al., 2021). And *Enterobacter*, *Sphingomonas*, *Pantomonas*, and *Methylobacterium* have also been reported to be prevalent during cigar fermentation (Zhang et al., 2023a; Zhou et al., 2020). Moreover, the rich microbial community in cigar tobacco leaves can promote the degradation and conversion of macromolecules like proteins, cellulose, and hemicellulose into small molecules such as alcohols, esters, aldehydes, and ketones through metabolic activities. This process enhances the color, physical properties, and aroma quality of cigars, while also assisting in reducing the production of harmful components like ammonia and nicotine (Banožić et al., 2020; Dai et al., 2020). For instance, *Bacillus* can produce small aromatic compounds by breaking down carotenoids (Dai et al., 2020), while *Pseudomonas* are effective in degrading nicotine during fermentation (Zhong et al., 2010; Liu et al., 2015; Wang et al., 2023). *Staphylococcus* has been reported to have a good performance in carbohydrate catabolism, amino acid transformation, protein hydrolysis and lipolysis, facilitating the degradation of branched-chain amino acids into methyl-branched-chain alcohols, aldehydes, carboxylic acids, and esters (Wang et al., 2024). Therefore, exploring the microbial communities and their metabolic functions during the processing of cigar tobacco leaves is crucial for understanding the quality formation mechanisms of tobacco leaves and also aids in developing and enhancing new products to meet consumer preferences. Previous research on microbial communities in cigar tobacco leaves has predominantly focused on the fermentation stage, characterizing microbial types and metabolic functions using cultivation and non-cultivation methods (Han et al., 2016; Ye et al., 2017). Regrettably, the microbial community and their metabolic functions during the air-curing process of cigar tobacco leaves remain unclear. Additionally, it is yet to be determined whether differences exist in microbial communities and functions among different parts of the tobacco leaves and if these differences have inherent connections with flavor compounds.

Therefore, this study initially analyze the microbial community structure and dynamic changes of cigar tobacco during the air-curing process by high-throughput sequencing. Moreover, the metabolic potential of microbial communities in different parts of tobacco leaves was predicted based on PICRUSt2. Subsequently, the volatile flavor components of different tobacco parts during air-curing were revealed by HS-SPME-GCMS, and the characteristic flavor components during air-curing were identified based on PLS-DA. Finally, statistical analyses were conducted to explore the potential correlations between microbial communities and flavor components formation.

## 2 Materials and methods

### 2.1 Sample collection

The tobacco leaves used in this study all obtained from the same batch of cigar tobacco leaves undergoing the air-curing process, provided by Hubei China Tobacco Industry Co., Ltd. Samples were taken randomly and uniformly from the upper and middle sections of the tobacco leaves at five stages of air-curing (fresh leaf, withering, yellowing phase, browning phase, and post-air-curing). After sampling, tobacco leaves from the same sections were mixed and placed in sterile bags. Each sampling was conducted three times for biological replication, and the samples were stored at −20°C. Specific sample information, including names, is presented in Table 1.

**Table 1:** Sample information of tobacco leaves in different parts during air-curing.

Period	Upper part	Middle part
Fresh leaf	CX10-T-0	CX10-M-0
Withering	CX10-T-1	CX10-M-1
Yellowing	CX10-T-2	CX10-M-2
Browning	CX10-T-3	CX10-M-3
Post-air-curing	CX10-T-4	CX10-M-4

### 2.2 DNA extraction

The tobacco leaves samples (5 g) were ground into powder in a pre-chilled mortar with liquid nitrogen and then mixed with 100 mL of sterile physiological saline solution (0.9% NaCl) in a 250 mL conical flask. The mixture was agitated on a shaker at 10°C and 220 rpm for 3 h. After filtering the tobacco debris through four layers of gauze, the microbial cells were pelleted by centrifugation at 4°C and 7,000 rpm for 15 min. The bacterial precipitate was collected. Total microbial DNA was extracted using the DNeasy PowerSoil Pro Kit, and the purity and concentration of DNA were assessed through agarose gel electrophoresis. Each sample underwent three biological replicates, and the extracted DNA samples were stored at −80°C for subsequent amplicon sequencing analysis.

### 2.3 DNA sequencing and functional prediction

The extracted DNA was used as a template, bacterial 16S rRNA V4–V5 region genes were amplified with bacterial sequence primers 515F (5’-GTGCCAGCMGCCGCGGTAA-3′) and 907R (5’-CCGTCAATTCMTTTRAGTTT-3′), while fungal ITS1 region genes were amplified with fungal sequence primers ITS1F (5’-CTTGGTTCATTTAGAGGAAGTAA-3′) and ITS2R (5’-GCTGCGTTCTTCATCGATGC-3′). The raw sequences were processed using QIIME2 software (Bolyen et al., 2019), which involved trimming low-quality regions, demultiplexing sample data based on barcodes, and removing barcodes and primer sequences to obtain preliminary quality-controlled raw data. The UCHIME Algorithm was used to detect and remove chimeric sequences, resulting in valid data. The valid data were clustered into Amplicon Sequence Variants (ASVs) at a 99% similarity level, and species classification annotations were performed using the q2-feature-classifier plugin. Predicted functional analysis of microbial community genes was conducted using PICRUSt2 (Douglas et al., 2020).

### 2.4 Identification of differential microorganisms

The Linear Discriminant Analysis Effect Size (LEfSe) method was employed to identify differential microorganisms in different tobacco leaf sections during the air-curing process, where microorganisms with an LDA score greater than 2 were considered as differential and characteristic microorganisms for the corresponding tobacco leaf sections (Jiao et al., 2018).

### 2.5 Volatile compound analysis

The tobacco leaf samples were dried in an oven at 40°C. Then, 1.5 g of the dried tobacco leaves were ground into powder and placed in a headspace vial for the determination of volatile compound content using Headspace Solid-Phase Microextraction combined with Gas Chromatography–Mass Spectrometry (HS-SPME-GC–MS). 2-Octanol at a concentration of 100 μg/μL was added as an internal standard. The SPME fiber assembly (50/30 μm DVB/CAR/PDMS, Supelco, United States) was used for extraction at 60°C for 30 min.

Volatile compounds were analyzed using a Gas Chromatography-Mass Spectrometer (7890A/5975C, Agilent Technologies, United States). The analysis conditions were as follows: the desorption time was set at 5 min; the column used was DB-5MS (30 m × 250 μm × 0.25 μm, Agilent Technologies, United States), with a column flow rate of 1 mL/min; the injection temperature was 250°C, starting at 40°C held for 2 min, then ramping up at a rate of 10°C/min to 250°C and held for 6 min. The ionization method used was Electron Impact (EI) at −70 eV; the transfer line and ion source temperatures were 280°C and 210°C, respectively. Mass spectral data were recorded in full-scan mode, scanning from m/z 33–400, with a solvent delay time of 3 min and a scan rate of 10 spectra per second. Data processing was performed using Chroma TOF 4.3X software and the LECO-Fiehn Rtx5 database, including raw peak extraction, data baseline filtering and calibration, peak alignment, deconvolution analysis, peak identification, and integration. Qualitative analysis of volatile flavor compounds was conducted by matching against the WILEY 8.0 and NIST14 libraries, and the relative content of volatile compounds was calculated using the area normalization method.

### 2.6 Statistical analysis and visualization

The *Spearman* correlation coefficients between tobacco leaf microorganisms and characteristic flavor components were calculated using the psych package in R software (Version 4.2.2), where *p* < 0.05 and *r* > 0.6 were defined as significant positive correlations, and *p* < 0.05 and *r* < −0.6 were defined as significant negative correlations. Gephi software (Version 0.9.4) and Cytoscape software (Version 3.9.1) were utilized for visualizing correlation networks (Xue et al., 2020). PCoA analysis of microbial communities was performed using the ade4 and ggplot2 packages in R software. The heatmap of volatile flavor compounds in cigar tobacco leaves was generated using the ggplot2 package in R software.

## 3 Results

### 3.1 Microbial community structure and dynamics in different tobacco leaf parts

**Figure 1:**
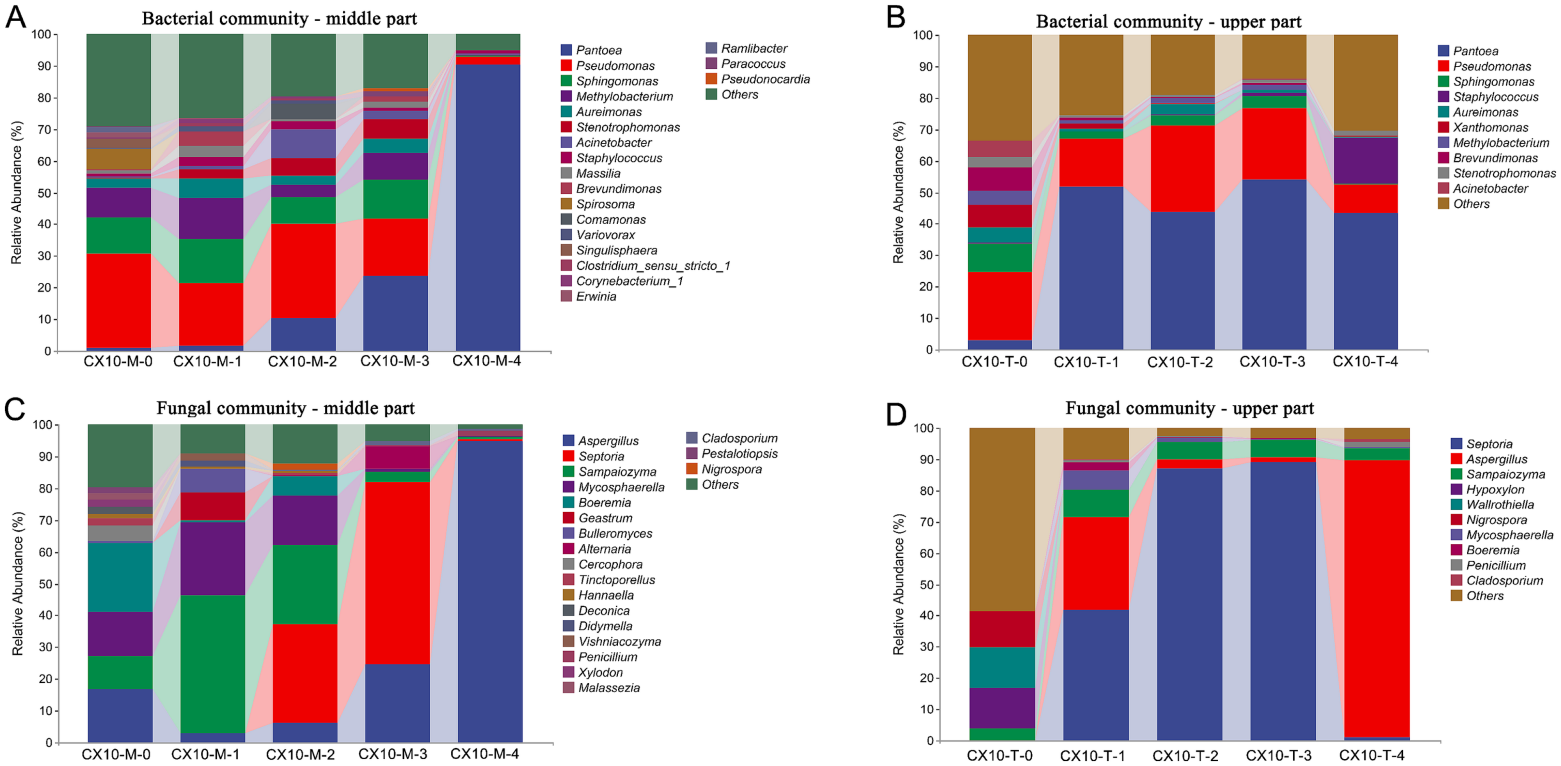
The microbial community structure in different parts of cigar tobacco leaves. **(A)** Bacterial community – middle part. **(B)** Bacterial community – upper part. **(C)** Fungal community – middle part. **(D)** Fungal community – upper part.

Cigar tobacco leaves are typically air-cured in an open environment where suitable temperature, humidity, and organic matter provide an optimal environment and substrate for microbial growth and metabolism (Zhong et al., 2023). Exploring the microbial community structure and dynamics during air-curing helps deepen our understanding of the material transformation mechanisms of tobacco. High-throughput sequencing was utilized to analyze the dynamic changes in microbial communities in different parts of cigar tobacco leaves during air-curing (Supplementary Figure S1 and Figure 1). In essence, at the level of bacterial phylum, *Proteobacteria* is the absolute dominant phylum in central and upper tobacco leaves, accounting for 70.3–97.4% of the relative abundance (Supplementary Figure S1). Followed by *Firmicutes*, which occupied a higher relative abundance in the fresh leaf (8.0%) and post-air-curing (15.1%) periods of the upper tobacco leaves, while the middle tobacco leaves were rich in the withering (7.6%) and yellowing (7.9%) stages. In terms of phyla, *Ascomycota* (35.8–97.9%) and *Basidiomycota* (3.9–64.1%) were dominant, with the sum of their relative abundance exceeding 95% in all samples (Supplementary Figure S1). Interestingly, the relative abundance of *Basidiomycota* increased obviously in both the middle part (64.1%) and upper part (11.1%) leaves in the withering stage (Supplementary Figure S1).

At the further microbial genus level, *Pseudomonas* (18.3–29.9%) and *Sphingomonas* (8.5–13.8%) were dominant bacterial genera in the middle part of leaves from the fresh stage to the browning stage, while *Pantoea* (1.7–90.4%) significantly increased from the yellowing stage, becoming the predominant bacterial genus by the end of air-curing (Figure 1A). Similarly, in the upper part of the leaves, *Pseudomonas* (9.0–27.6%), *Sphingomonas* (0.5–9.3%), and *Pantoea* (3.0–54.1%) were dominant bacterial genera throughout the air-curing process. However, the relative abundance of *Pantoea* (3.0–54.1%) notably increased starting from the withering stage and became the most abundant genus (Figure 1B). Regarding fungal genera, the fungal community structures differed significantly in different parts of the tobacco leaves (Figures 1C,D). In the mid-section of the leaves, the relative abundance of the genus *Aspergillus* (24.8–94.9% in middle part and 1.9–88.9% in upper part) sharply increased after the browning stage, and becoming the predominant fungus in post-air-curing stage, with a relative abundance more than 90% (Figure 1C). Of particular interest, the genera *Septoria* and *Sampaiozyma* exhibited higher abundance from withering to browning stages, but nearly disappeared post-air-curing, possibly due to the gradual reduction of leaf moisture during the air-curing process, indicating a lower environmental stress resistance of these microorganisms.

### 3.2 Microbial community diversity analysis and differential microbial identification

**Figure 2:**
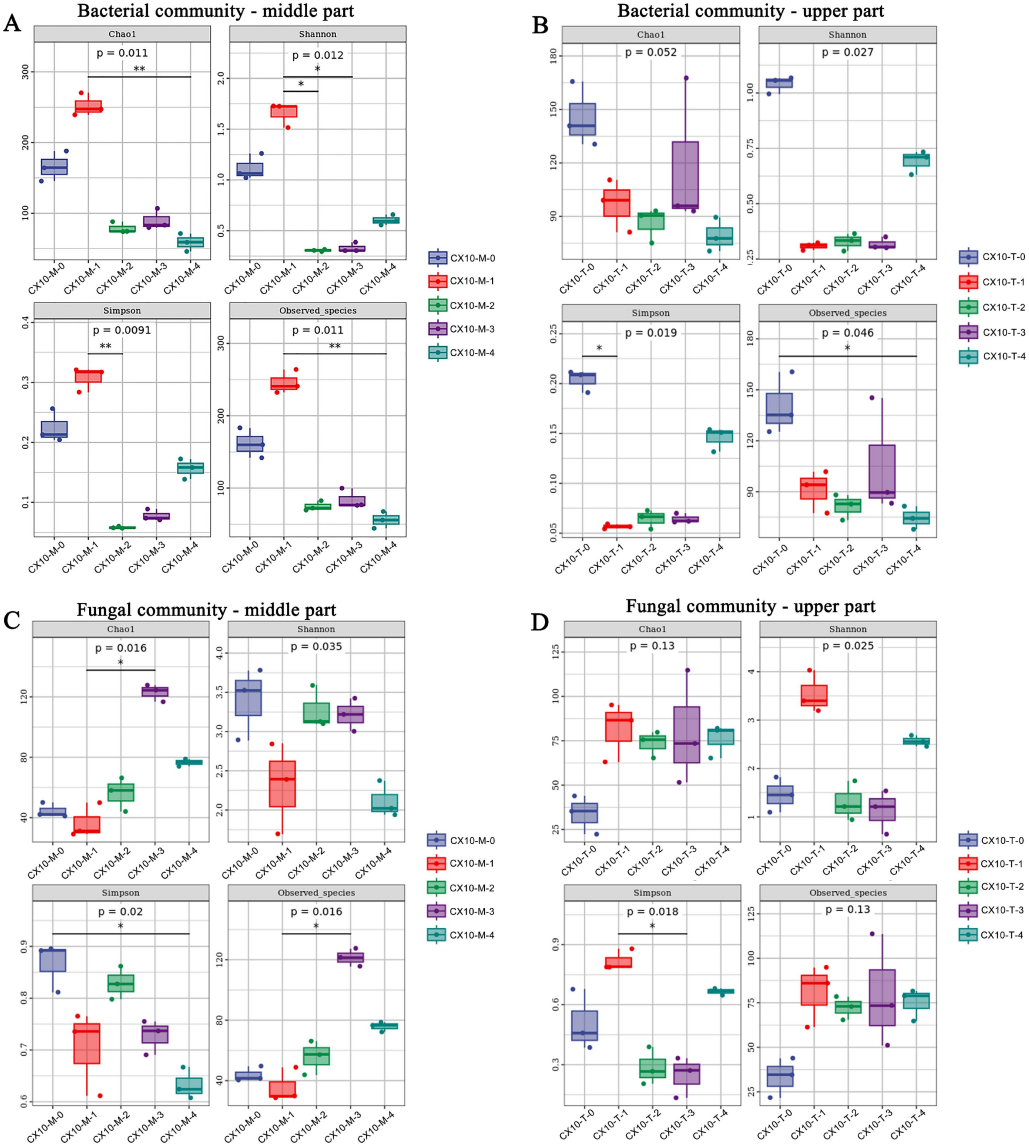
The alpha diversity of microbial communities in different parts of cigar tobacco leaves. **(A)** Bacterial community – middle part. **(B)** Bacterial community – upper part. **(C)** Fungal community – middle part. **(D)** Fungal community – upper part.

The analysis of microbial community diversity aims to investigate the differences in microbial populations among different tobacco leaf parts during the air-curing process (Figure 2). In terms of alpha-diversity, the Chao1, Shannon, Simpson, and observed species of bacteria communities in the mid-section of leaves during the withering stage were significantly higher than other stages, indicating that the highest richness and species diversity (Figure 2A). Conversely, for the upper part of the leaves, the richness and diversity of bacterial communities were highest during the fresh leaf stage, followed by the browning stage and post-air-curing period (Figure 2B). As for fungal communities, the species richness in the mid-section leaves was highest during the browning stage, while the diversity was highest during the fresh leaf stage (Figure 2C). For the upper part of the leaves, the diversity of fungal communities was highest during the withering stage, while the richness was lowest during the fresh leaf stage (Figure 2D).

**Figure 3:**
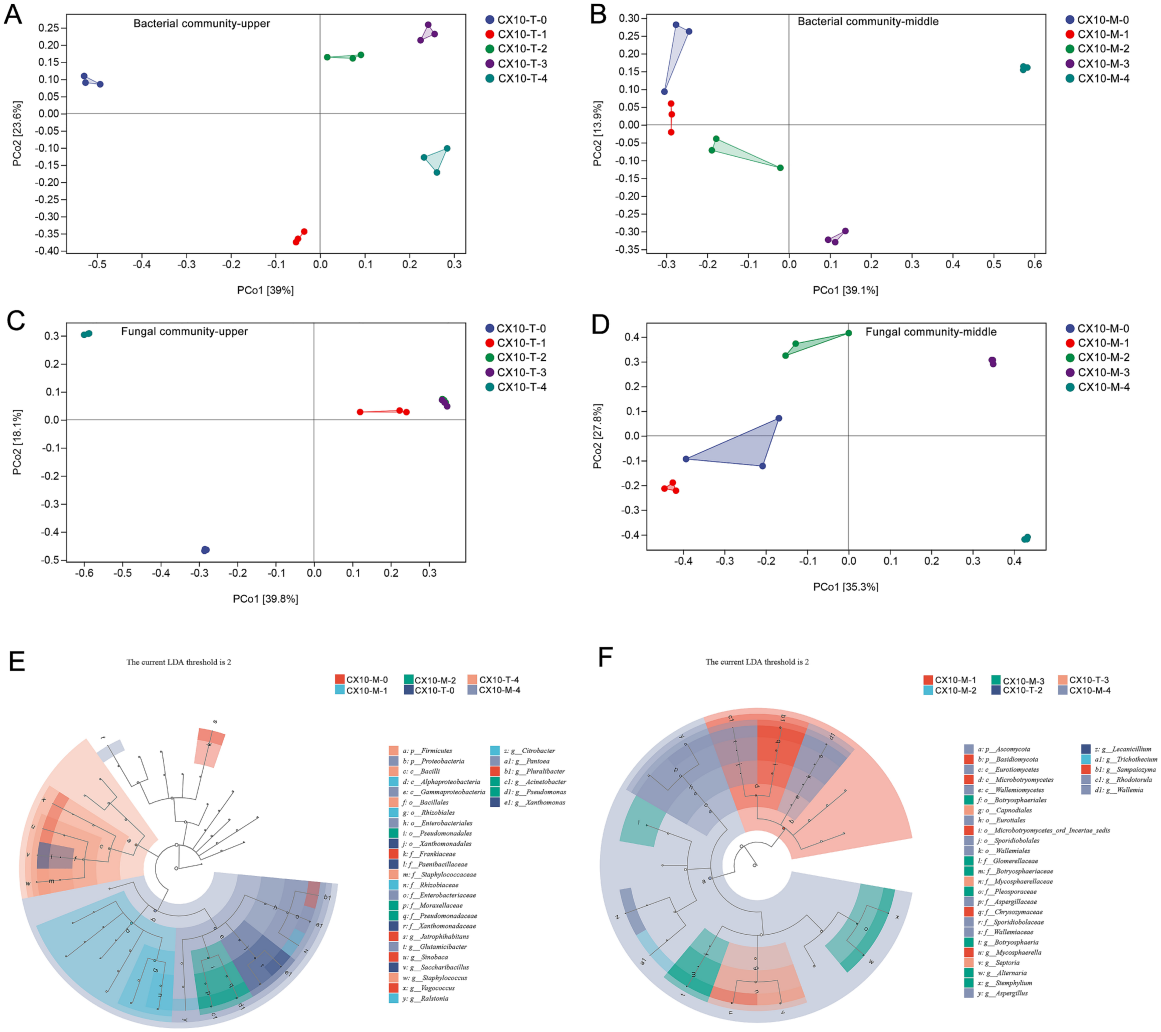
The beta diversity of microbial communities and identification of differential microbial in different parts of cigar tobacco leaves. **(A)** Bacterial community – upper part. **(B)** Bacterial community – middle part. **(C)** Fungal community – upper part. **(D)** Fungal community – middle part. **(E)** Differential bacteria between middle and upper tobacco leaves. **(F)** Differential fungi between middle and upper tobacco leaves.

Principal coordinates analysis (PCoA) was conducted to assess the beta-diversity of microbial communities at the genus level in different tobacco leaf parts during the air-curing process. As shown in Figure 3, significant changes in bacterial communities of the upper part of the leaves were observed in the early stages of air-curing, but the extent of change decreases in subsequent stages (Figure 3A). In contrast, the bacterial communities in the mid-section of leaves show minimal variation in the early stages of air-curing, with significant changes occurring only after the yellowing stage (Figure 3B). In terms of fungal communities, obviously changes were evident in the upper part of the leaves during the withering stage and post-air-curing period (Figure 3C). The differences in fungal communities of the mid-section of leaves were minor in the early stages of air-curing, but substantial changes occurred after the yellowing stage, intensifying further after the browning stage (Figure 3D).

In order to explore the differential microorganisms in tobacco leaves during the curing stage, microbial genera with an LDA score > 2 were identified as differential and characteristic microorganisms of the air-curing process using LEfSe (Figures 3E,F). Specifically, the differential bacteria genera between the middle and upper part of tobacco leaves during curing include *Jatrophihabitans*, *Glutamicibacter*, *Sinobaca*, *Saccharibacillus*, *Staphylococcus*, *Vagococcus*, *Ralstonia*, *Citrobacter*, *Pantoea*, *Acinetobacter*, *Pseudomonas* and *Xanthomonas* (Figure 3E). Moreover, the differential fungal genera between the middle and upper part mainly involved *Botryosphaeria*, *Mycosphaerella*, *Septoria*, *Alternaria*, *Siemphyiium*, *Aspergillus*, *Trichothecium*, *Rhodotorula*, and *Wallemia* (Figure 3F).

### 3.3 Prediction of microbial metabolic function

**Figure 4:**
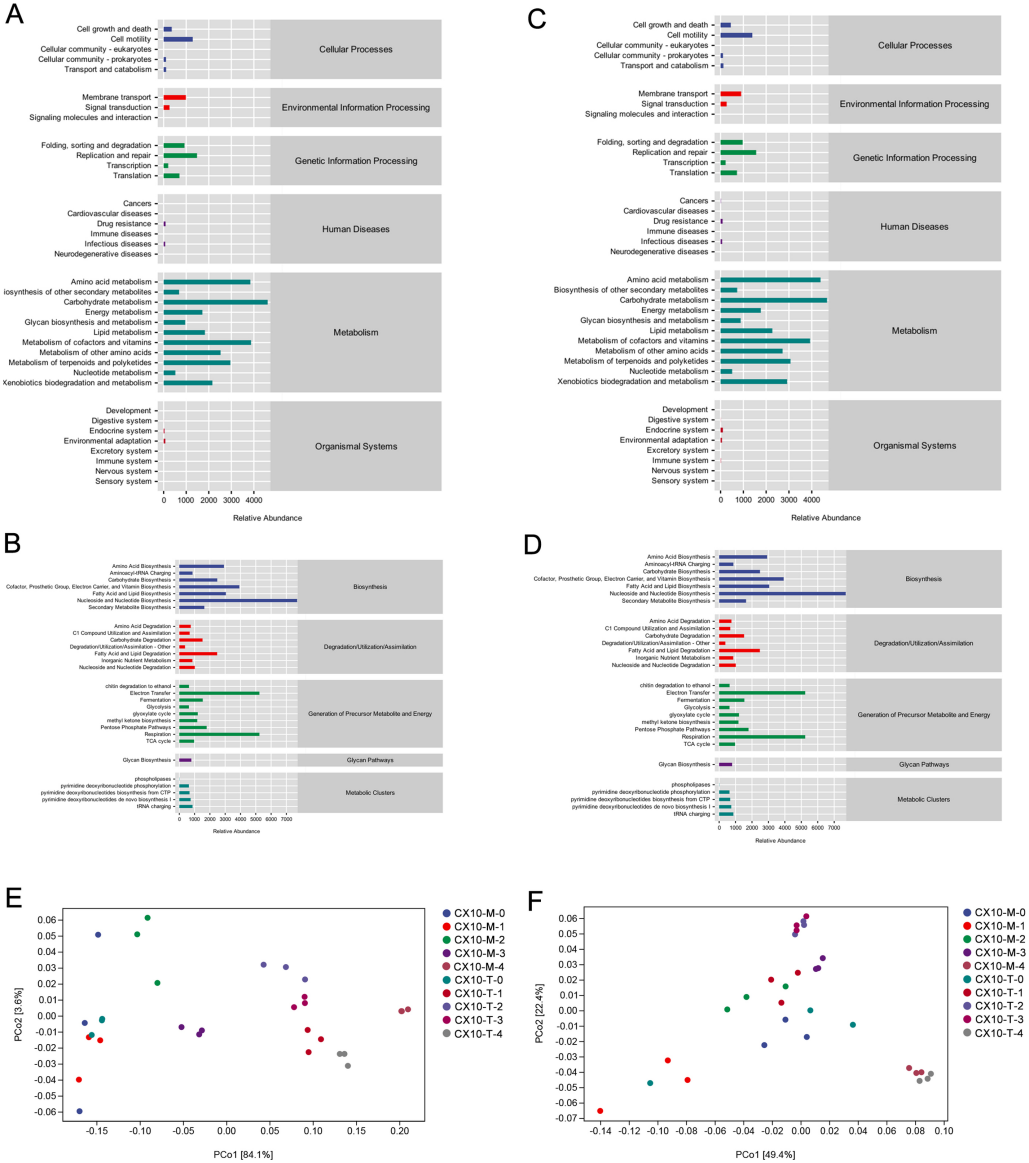
Prediction of microbial community metabolic function. **(A)** Bacterial community – upper part. **(B)** Fungal community – upper part. **(C)** Bacterial community – middle part. **(D)** Fungal community – middle part. Difference analysis of metabolic function of bacterial communities **(E)** and fungal communities **(F)** in different tobacco leaf parts.

The microbial metabolic function of different tobacco leaf parts during the air-curing process was predicted using PICRUSt2 software, and the functional differences in microbial communities between various leaf sections were analyzed (Figure 4). As depicted in the Figures 4A,B, the microbial community in the upper part of the leaves primarily engages in carbohydrate metabolism, amino acid metabolism, cofactor and vitamin metabolism, terpenoid and polyketide metabolism, as well as the biodegradation and metabolism of exogenous substances throughout the air-curing process. The microbial community functions in the middle part of leaves resemble those of the upper part (Figures 4C,D), focusing on pathways like carbohydrate metabolism, amino acid metabolism, as well as cofactor and vitamin metabolism. Notably, in the microbial community functions of the mid-section, the abundance of pathways related to carbohydrate and amino acid metabolism was significantly higher than in the upper part, indicating a potentially stronger utilization and transformation capability of sugars and amino acids by the mid-section microbial community, while the lipid metabolism capacity of microbes in the upper part was comparatively weaker.

The differential microbial community functions in different leaf sections during the air-curing process were assessed through PCoA (Figures 4E,F). For the upper part of the leaves, both bacterial and fungal community functions show substantial differences from the fresh leaf stage to the yellowing stage, indicating the yellowing stage as a critical transition point for microbial community functions in the upper part of the leaves. In contrast, significant differences in microbial community functions of the mid-section of leaves were observed between the post-air-curing stage and other periods, suggesting the post-air-curing period as a pivotal transformation stage for the mid-section leaves (Figures 4E,F). Furthermore, we observed that microbial community functions in both bacterial and fungal communities in different leaf parts are more similar during the yellowing and browning stages, may implying a gradual and orderly change process in tobacco leaf quality during this period.

### 3.4 The dynamics of volatile flavor components in different tobacco leaf parts

**Figure 5:**
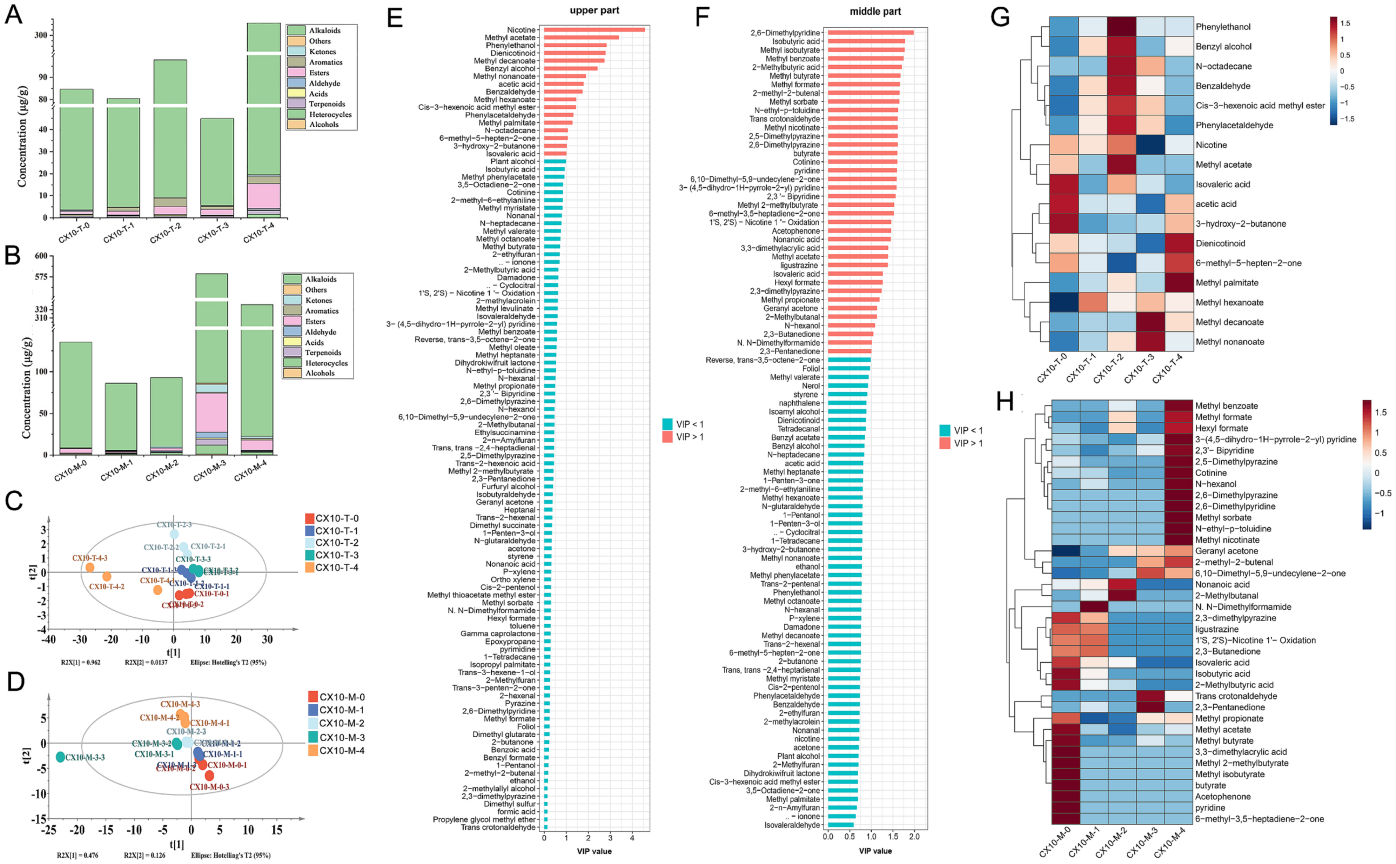
Analysis of volatile flavor compounds. Columnar accumulations of volatile substance content in upper tobacco **(A)** and middle tobacco **(B)**. PLS-DA score plot **(C)**, VIP values **(E)** and heatmap **(G)** of volatile flavor compounds in upper tobacco leaves. PLS-DA score plot **(D)**, VIP values **(F)** and heatmap **(H)** of volatile flavor compounds in middle tobacco leaves.

Through HS-SPME-GC–MS, 92 and 104 volatile flavor compounds were identified in the upper and middle parts of tobacco leaves, respectively (the specific concentrations of volatile compounds are shown in Supplementary Tables S1, S2). The variations in the content of different categories of substances during the air-curing process as shown in Figures 5A,B. Among them, alkaloids represented the most predominant volatile compounds in tobacco leaves, primarily including nicotine and its degradation products, such as cotinine and dienicotinoid. Notably, the content of alkaloids in the upper part of tobacco leaves significantly increased in post-air-curing period, reaching 288.0 μg/g (Figure 5A), while in the middle part, the content of alkaloids rose during the browning stage and post-air-curing period, reaching a final concentration of 303.8 μg/g (Figure 5B). As the air-curing progressed, the content of various substances in the upper part of tobacco leaves showed an increasing trend, especially esters, which increased from 1.4 μg/g in the fresh leaf stage to 11.4 μg/g of post-air-curing period. The content of various substances in the middle part of the leaves mainly peaked during the browning stage and experienced a decrease at the post-air-curing period.

The PLS-DA model was established to reveal the characteristic volatile compounds of tobacco leaves from different parts during the air-curing process. As illustrated in the PLS-DA score plot, there were clear differences in the volatile flavor characteristics of cigar tobacco leaves throughout the air-curing process (Figures 5C,D). Seventeen and 38 compounds were identified from the upper and middle parts of the leaves showed significant changes (VIP > 1) during the air-curing process and were considered characteristic volatile compounds for these two parts, respectively (Figures 5E,F). As for specific compounds, characteristic compounds in the upper leaves during the fresh leaf phase include isovaleric acid, acetic acid, and 3-hydroxy-2-butanone, while the yellowing phase is characterized mainly by alcohols and aldehydes such as phenylethanol, benzyl alcohol, phenylacetaldehyde, and benzaldehyde (Figure 5G). Esters like methyl decanoate and methyl nonanoate mainly accumulate during the browning phase, with dienicotinoid and methyl palmitate marking the post-air-curing period. For the middle part of the leaves (Figure 5H), characteristic compounds were primarily concentrated at the fresh leaf stage and the post-air-curing period process. Flavor components like isovaleric acid, isobutyric acid, 2-methylbutyric acid, methyl propionate, and methyl butyrate were enriched in the fresh leaf stage and decrease as they transform throughout the air-curing process, whereas pyridines, pyrazines, and esters significantly increase in the post-air-curing period.

### 3.5 Correlation analysis between differential microorganisms and characteristic volatile flavor compounds

**Figure 6:**
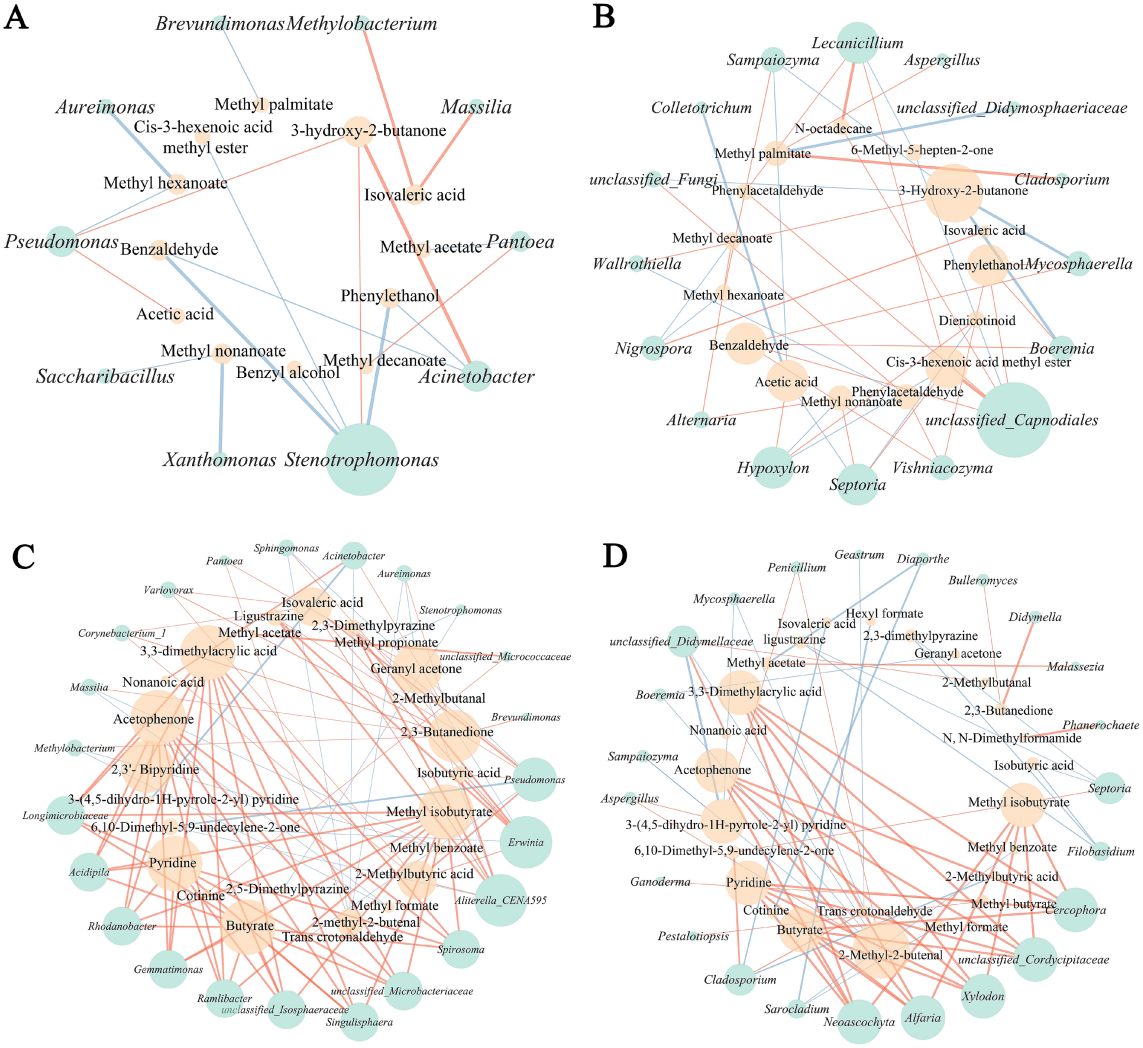
Correlation analysis between microorganisms and volatile flavor compounds. **(A)** Bacteria and flavor compounds of upper part. **(B)** Fungi and flavor compounds of upper part. **(C)** Bacteria and flavor compounds of middle part. **(D)** Fungi and flavor compounds of middle part.

The potential correlation between differential microbes and characteristic volatile flavor compounds of different tobacco leaf parts were examined based on *Spearman* correlation coefficient (Figure 6). The correlation network of differential microorganisms and flavor compounds in the upper tobacco leaves as shown in Figures 6A,B. In this network, *Pseudomonas* showed a significant positive correlation with the production of 3-hydroxy-2-butanone and acetic acid, while the *Stenotrophomonas* exhibited a clearly negative correlation with the formation of benzaldehyde, phenylethanol, benzyl alcohol and cis-3-hexenoic acid methyl ester, implying a possible adverse role in the formation of tobacco flavor (Figure 6A). As for fungi, unclassified_*Capnodiales* demonstrated a significant positive correlation with various flavor substances, potentially enhancing the generation of *Cis*-3-hexenoic acid methyl ester, benzaldehyde, *N*-octane, phenylethanol, and other flavor compounds, significantly contributing to the shaping of tobacco flavor (Figure 6B). Additionally, most differential fungi appeared to promote the production of aroma substances such as benzaldehyde, phenylethanol, methyl palmitate, acetic acid, and nonanoic acid, enriching the floral and fruity aroma characteristics of tobacco. Notably, 3-hydroxy-2-butanone exhibited a negative correlation with various differential fungi but a positive correlation with various differential bacteria, indicating distinct functional differences among different microbial groups in the formation of tobacco flavor.

The correlation network of differential microorganisms and flavor compounds in the middle tobacco leaves represented in Figures 6C,D. The statistical relationships between differential microorganisms and flavor compounds in the middle tobacco leaves appeared to be more complex and diverse compared to the upper tobacco leaves. Multiple differential microorganisms showed similar flavor-related features with flavor compounds. For instance, bacteria such as *Rhodanobacter*, *Gemmatimonas*, *Ramlibacter*, *Longimicrobiaceae*, *Singulisphaera*, *Erwinia*, *Aliterella_*CENA595, unclassified_*Microbacteriaceae*, and unclassified_*Isosphaeraceae* all exhibited a significant positive correlation with flavor substances like 3,3-dimethylacrylic acid, acetophenone, 2,3-dihydropyridine, pyridine, butyrate, methyl isobutyrate, 2,3-butanedione, and geranyl acetone (Figure 6C). In terms of fungi, *Neoascochyta*, *Alfaria*, *Xylodon*, unclassified_*Cordycipitaceae* and *Cerophora* potentially cooperated in promoting the generation of substances like 3,3-dimethylacrylic acid, acetophenone, pyridine, butyrate, 2-methyl-2-butenal, and methyl isobutyrate (Figure 6D), leading to the production of these rich aroma components during the browning and post-air-curing period. These correlation insights provide valuable understanding of the intricate interactions between microbial communities and flavor formation across different parts of tobacco leaves during the curing process.

## 4 Discussion

Cigar tobacco has a long history of cultivation and processing (Haustein and Groneberg, 2010). Unlike the processing method for cigarette tobacco, cigar tobacco must undergo air-curing after harvesting before proceeding to agricultural and industrial fermentation. This method is one of the primary reasons for the unique flavor and aroma of cigars tobacco (He et al., 2013). The air-curing process typically takes place in an open environment, where suitable environmental conditions and rich organic matter in the leaves promote the growth and metabolism of microorganisms, thus affecting the quality of the tobacco leaves. Therefore, this study analyzed the dynamics of microbial communities and flavor compositions in different parts of cigar tobacco leaves during the air-curing process, revealing characteristic microbial communities and key volatile compounds. Additionally, it further explored the potential relationships between the two, aiming to elucidate the microbial composition in different parts of tobacco leaves and their impact on the formation of cigar flavors during air-curing.

High-throughput sequencing was employed to analyze the microbial communities in tobacco leaves during the curing process. Diversity analysis results indicated that the richness and species diversity of bacterial communities in the middle and upper tobacco leaves were higher in the early stages of curing, possibly due to the gradual evaporation of moisture in the leaves as the curing process progressed, making the environment less conducive for the growth of most microorganisms (Niu et al., 2024). *Pseudomonas*, *Sphingomonas*, and *Pantoea* were identified as dominant microorganisms in both the middle and upper tobacco leaves during curing. It is well known that *Sphingomonas* possesses excellent macromolecule degradation capabilities, being able to degrade starch, cellulose, proteins, and other macromolecules, potentially contributing to the formation of cigar flavors (Chen et al., 2024; Tao et al., 2022). The abundance of *Pantoea* gradually increased throughout the curing process, likely due to its good environmental adaptability. Previous studies have shown that *Pantoea* is commonly found in traditional fermentation environments for rice wine (Chen et al., 2022), Baijiu (Luo et al., 2023), and vinegar (Li et al., 2022), exhibiting good environmental stress resistance and close associations with the formation of various flavor compounds. *Aspergillus* and *Septoria* were the predominant fungi during air curing of cigar leaves. Previous studies indicate that *Aspergillus* is a dominant fungus during the pile fermentation process, producing enzymes like proteases and amylases that enhance aroma compound production, thus improving cigar quality (Wu et al., 2023).

In terms of flavor compounds, 92 and 102 volatile compounds were identified in tobacco leaves during the air-curing process using HS-SPME-GC–MS, respectively. Furthermore, 17 and 38 significantly changed characteristic flavor components were identified in the upper and middle tobacco leaves during air-curing through PLS-DA, respectively. Alcohols primarily begin to form after the withering phase, providing floral, fruity, and sweet aromas to the leaves and softening their harshness (Li et al., 2022). It is worth mentioning that esters compounds were mostly formed during the browning stage, and they often possess floral and fruity aromas, capable of softening the harshness of the smoke and endowing tobacco with a pleasant aromatic sensation (Zhang et al., 2024). Heterocyclic flavor components like pyridines, pyrroles, and pyrazines mostly enrich toward the end of the post-air-curing process, potentially enhancing nutty and bakery aromas in the leaves (Zheng et al., 2022). Pyrazines, often carrying nutty and roasted aromas, might help enhance the richness of cigar tobacco aroma (Zheng et al., 2022). Alkaloids are the most significant volatile compounds in tobacco, with a substantial increase starting in the browning phase. Excessive alkaloid content can intensify the sharpness and bitterness of cigar smoke, while too low levels might result in insufficient vigor and bland aroma (Wu et al., 2022; Zelinkova and Wenzl, 2021). It is important to note that due to the addictive nature of nicotine, excessive smoking of tobacco can sharply increase the risk of lung, throat, and oral cancers (Gould, 2023; Murphy, 2021). A previous research has reported the nicotine content in cigar tobacco is relatively lower compared to other tobacco products (Wu et al., 2023).

Microorganisms play a role in improving the sensory characteristics of tobacco, such as color, aroma, and flavor. Differences in microbial community structure and metabolic functions in different parts may be the main reason for variations in volatile flavor compounds (Jin et al., 2019; Song et al., 2017; Yin et al., 2019). PICRUSt2 was used to predict the microbial community functions in different parts of cigar tobacco leaves during the air-curing process. It was found that the abundance of metabolic pathways related to carbohydrate and amino acid metabolism in the microbial community of the middle part was significantly higher than in the upper leaves, potentially leading to more intense substance transformation activities in the middle leaves, resulting in significant changes in flavor components during the air-curing process. Moreover, the functional diversity of microbial communities in the post-air-curing differed significantly from that of other periods, suggesting that this period may be crucial for important changes in tobacco quality during air-curing process.

Finally, this study also explored the correlation between characteristic microorganisms and flavor compounds through network analysis. It is worth mentioning that the molecular network correlation of central tobacco leaves is more complex and diverse compared to upper tobacco leaves, indicating that the microorganisms in central tobacco leaves may play a more significant metabolic role during the air-curing process, which is in line with the predicted results of community metabolic functions. There are more positively correlated features between bacterial communities and flavor characteristics, consistent with previous research findings (Liu et al., 2021; Zheng et al., 2022). Our previous study reported that *Acinetobacter*, *Sphingomonas*, *Solibacillus*, and *Lysinibacillus* are the primary microbial producers of carbonyl compounds in cigars (Zheng et al., 2022). Similarly, *Acinetobacter* and *Sphingomonas* are identified in this study as characteristic microorganisms of upper and central tobacco leaves, positively correlated with the formation of carbonyl compounds such as 3-hydroxy-2-butanone and 2,3-butanedione. Furthermore, *Pseudomonas* is significantly positively correlated with various flavor substances during the curing process, potentially enhancing the formation of flavor characteristics of tobacco leaves at that stage. In contrast, *Stenotrophomonas* shows a negative correlation with aromatic substances such as benzaldehyde, phenylethanol, benzyl alcohol, and *cis*-3-hexenoic acid methyl ester, suggesting a potential adverse impact on flavor development during the air-curing process. These results indicate that bacteria may dominate key microbial communities responsible for the formation of tobacco flavor quality during different processing stages, and differences in microbial communities and their metabolic functions are likely the primary reasons for variations in tobacco flavor compounds (Han et al., 2016; Su et al., 2011).

In summary, this study investigated the microbial community composition and dynamics of different parts of cigar tobacco leaves during the curing process, revealing characteristic microorganisms and flavor components of tobacco leaves at different stages. Through community function prediction and correlation analysis, it elucidated the potential roles played by microorganisms of cigar tobacco leaf. Overall, these research findings contribute to a deeper understanding of the mechanisms underlying flavor changes during the cigar tobacco leaf air-curing process, and aid in the selection of functional microorganisms for enhancing or stabilizing the quality of tobacco leaves in subsequent processes.

## Data Availability

The original contributions presented in the study are included in the article/Supplementary material, further inquiries can be directed to the corresponding authors.
