# Supplementary material for: The dynamics of microbial community structure and metabolic function in different parts of cigar tobacco leaves during air-curing
Source: Front Microbiol. 2024 Dec 12;15:1438566. doi: 10.3389/fmicb.2024.1438566 (PMC11669699; doi:10.3389/fmicb.2024.1438566)
Supplement: Supplementary file 1 [file Data_Sheet_1.docx]

Supplementary materials

**Figure S1:** Microbial community phylum levels in different parts of cigar tobacco leaves.

**Table S1:** The contents of volatile flavor compounds in upper part of tobacco leaves (ug/g)

**Table S2:** The contents of volatile flavor compounds in middle part of tobacco leaves (ug/g)

**
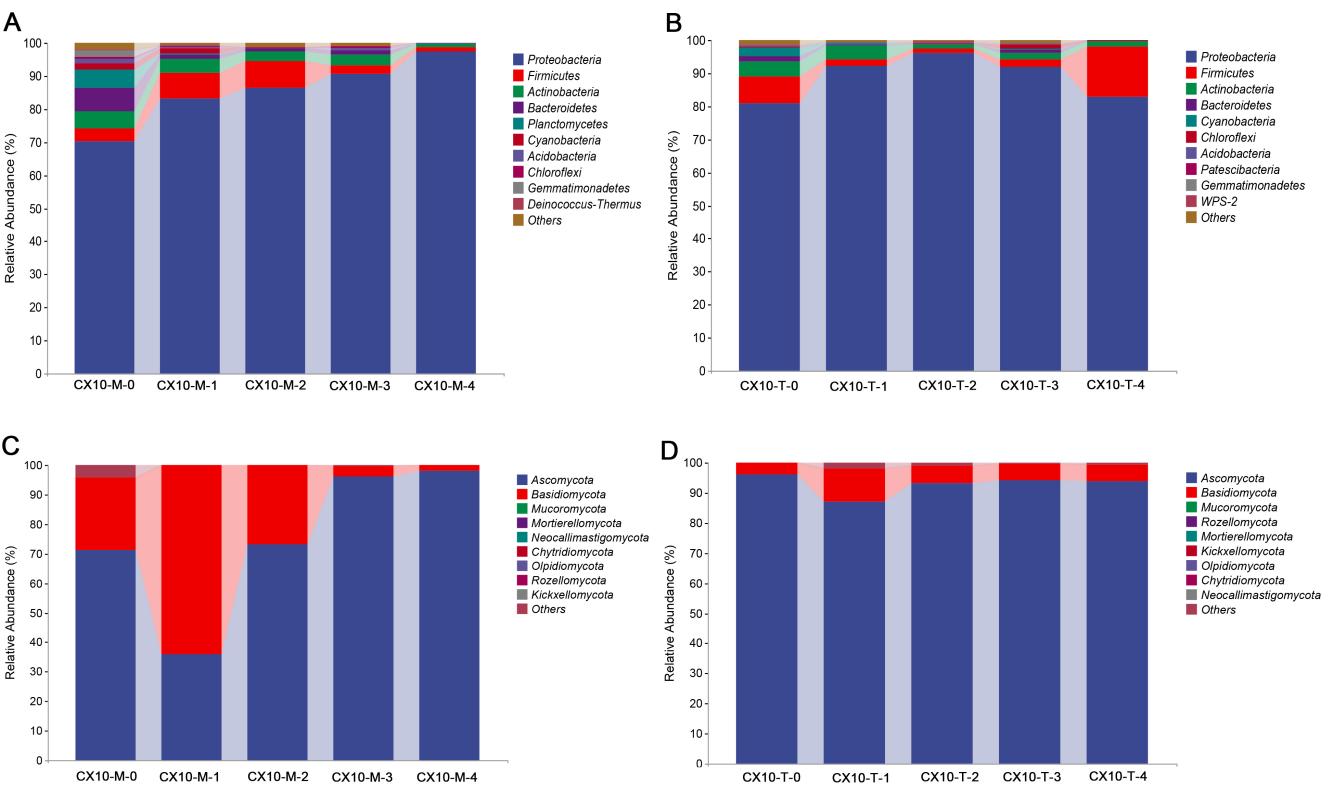
**

**Figure S1: Microbial community phylum levels in different parts of cigar tobacco leaves.**Bacterial phylum levels in the middle (A) and upper (B) parts of tobacco leaves. Fungal phylum levels in the middle (C) and upper (D) parts of tobacco leaves.

**Table S1: The contents of volatile flavor compounds in upper part of tobacco leaves (ug/g).**

| **Flavor compounds** | **Group** | **CX10-T-0** | **CX10-T-1** | **CX10-T-2** | **CX10-T-3** | **CX10-T-4** |
| --- | --- | --- | --- | --- | --- | --- |
| N-hexanol | Alcohols | - | - | - | - | 0.0628 |
| Cis-2-pentanol alcohol | Alcohols | 0.0180 | 0.0126 | 0.0023 | 0.0118 | - |
| 2-methylallyl alcohol | Alcohols | - | - | - | - | 0.0067 |
| Trans-3-hexen-1-ol | Alcohols | 0.0043 | 0.0082 | - | 0.0031 | - |
| 1-Pentene-3-ol | Alcohols | 0.0154 | 0.0027 | - | - | 0.0055 |
| ethanol | Alcohols | 0.0169 | 0.0163 | 0.0229 | 0.0148 | 0.0790 |
| 1-pentanol | Alcohols | - | - | - | - | 0.0324 |
| Leaf alcohol | Alcohols | 0.0098 | 0.0151 | 0.0232 | 0.0132 | 0.0079 |
| Furfuryl alcohol | Alcohols | - | 0.0073 | 0.0193 | - | - |
| 2,6-dimethylpyridine | Heterocycls | - | - | - | 0.0026 | 0.0612 |
| 2,6-dimethylpyrazine | Heterocycls | - | - | - | - | 0.0775 |
| 3- (4,5-dihydro-1H-pyrrole-2-yl) pyridine | Heterocycls | 0.1560 | 0.0737 | 0.1130 | 0.0712 | 0.7369 |
| 2,5-dimethylpyrazine | Heterocycls | - | - | - | - | 0.0673 |
| pyrimidine | Heterocycls | - | - | 0.0101 | - | - |
| Pyrazine | Heterocycls | - | - | 0.0041 | - | - |
| 2-ethylfuran | Heterocycls | 0.0640 | 0.0199 | 0.0158 | 0.0096 | 0.0307 |
| 2-n-pentyl furan | Heterocycls | 0.0700 | 0.0485 | 0.0404 | 0.0242 | 0.0795 |
| 2-methylfuran | Heterocycls | 0.0055 | 0.0129 | 0.0277 | 0.0160 | 0.1054 |
| 2,3 '- bipyridine | Heterocycls | 0.0354 | - | - | 0.0175 | 0.2817 |
| 2,3-dimethylpyrazine | Heterocycls | - | - | - | - | 0.0066 |
| Ketamine | Alkaloids | 0.0531 | 0.0920 | - | 0.0139 | 0.2304 |
| Nicotine Diene | Alkaloids | 1.1757 | 1.0459 | 0.7576 | 0.4509 | 0.8583 |
| 1'S, 2'S) - Nicotine 1 '- Oxidation | Alkaloids | 0.0319 | - | - | - | - |
| nicotine | Alkaloids | 79.5535 | 74.2992 | 87.9170 | 38.9243 | 286.9184 |
| Plant alcohol | Terpenoids | 0.0643 | 0.2188 | 0.1985 | 0.2210 | 0.6336 |
| Dihydrokiwilactone | Terpenoids | 0.1291 | 0.1767 | 0.1574 | 0.1580 | 0.6087 |
| Geranyl acetone | Terpenoids | 0.0151 | 0.0234 | 0.0164 | 0.0121 | 0.0560 |
| β - Violet Ketone | Terpenoids | 0.2850 | 0.2568 | 0.2185 | 0.1601 | 0.4651 |
| Nonanoic acid | Acids | 0.0105 | - | - | - | - |
| 2-methylbutyric acid | Acids | 0.0399 | 0.0144 | 0.0359 | 0.0093 | - |
| Trans-2-hexenoic acid | Acids | - | 0.0129 | 0.0078 | - | - |
| Isovaleric acid | Acids | 0.0611 | - | 0.0418 | 0.0041 | - |
| formic acid | Acids | - | - | 0.0014 | - | - |
| acetic acid | Acids | 0.5125 | 0.2722 | 0.2795 | 0.1255 | 0.3171 |
| Isobutyric acid | Acids | 0.0397 | - | 0.0583 | - | - |
| N-pentanal | Aldehydes | - | 0.0104 | 0.0093 | 0.0173 | 0.0044 |
| 2-methyl-2-butyraldehyde | Aldehydes | - | - | - | - | 0.0092 |
| Heptanal | Aldehydes | 0.0114 | - | - | - | - |
| Trans crotonaldehyde | Aldehydes | - | - | - | 0.0016 | - |
| Nonanal | Aldehydes | 0.0586 | 0.0956 | 0.0939 | 0.1424 | 0.2562 |
| Anti-2,4-heptadienal | Aldehydes | 0.0123 | 0.0032 | - | 0.0169 | 0.0319 |
| β - cyclic citral | Aldehydes | - | - | - | 0.0292 | - |
| 2-Hexenal | Aldehydes | 0.0096 | - | - | - | - |
| Isovaleraldehyde | Aldehydes | 0.0228 | 0.0375 | 0.0515 | 0.0158 | 0.0133 |
| N-hexanal | Aldehydes | 0.0337 | 0.0246 | 0.0138 | 0.0438 | 0.1028 |
| Trans-2-hexenal | Aldehydes | 0.0226 | 0.0117 | 0.0175 | 0.0240 | 0.0668 |
| Isobutyraldehyde | Aldehydes | - | 0.0014 | 0.0182 | - | - |
| 2-methylacrylaldehyde | Aldehydes | - | 0.0292 | 0.0021 | 0.0381 | 0.0753 |
| 2-methylbutanal | Aldehydes | 0.0044 | 0.0124 | 0.0221 | - | - |
| Benzyl formate | Esters | - | 0.0060 | 0.0111 | 0.0065 | - |
| Dimethyl succinate | Esters | - | - | 0.0201 | - | 0.0216 |
| Methyl caproate | Esters | 0.0174 | 0.2656 | 0.1910 | 0.2306 | 0.6163 |
| Methyl heptanoate | Esters | - | 0.0102 | - | 0.0329 | 0.0596 |
| Methyl formate | Esters | - | 0.0070 | 0.0157 | 0.0025 | 0.0191 |
| Methyl decanoate | Esters | 0.1456 | 0.3282 | 0.3313 | 1.0569 | 2.9518 |
| Methyl octanoate | Esters | - | 0.0425 | 0.0550 | 0.0804 | 0.1004 |
| Dimethyl glutarate | Esters | - | - | 0.0062 | - | - |
| Methyl palmitate | Esters | 0.2421 | 0.3826 | 0.5540 | 0.3747 | 3.5714 |
| Methyl oleate | Esters | - | - | - | - | 0.0326 |
| Methyl myristate | Esters | - | - | - | 0.0543 | 0.8748 |
| Cis-3-hexenoic acid methyl ester | Esters | 0.0063 | 0.2215 | 0.3531 | 0.2085 | 0.0554 |
| Isopropyl palmitate | Esters | 0.0040 | 0.0065 | 0.0058 | 0.0040 | - |
| Methyl sorbate | Esters | - | - | 0.0152 | - | - |
| Methyl thioacetate | Esters | - | 0.0098 | 0.0211 | 0.0149 | - |
| Methyl nonanoate | Esters | 0.0080 | 0.1152 | 0.2419 | 0.4233 | 0.6722 |
| Methyl Propionate | Esters | 0.0099 | 0.0066 | 0.0703 | 0.0089 | 0.1829 |
| Butyrate methyl ester | Esters | 0.0370 | - | 0.0334 | 0.0096 | 0.0632 |
| Methyl valerate | Esters | - | 0.0467 | - | 0.0564 | - |
| Methyl levulinate | Esters | - | - | 0.0407 | - | - |
| Hexyl formate | Esters | - | - | - | - | 0.0299 |
| Gamma Caprolactone | Esters | - | - | 0.0085 | - | - |
| Methyl acetate | Esters | 0.8622 | 0.1310 | 1.6379 | 0.1154 | 2.1767 |
| Methyl 2-methylbutyrate | Esters | - | - | 0.0222 | - | 0.0160 |
| styrene | Aromatics | 0.0110 | 0.0015 | - | 0.0034 | 0.0077 |
| Benzyl alcohol | Aromatics | 0.0971 | 0.4678 | 0.7563 | 0.1872 | 0.1930 |
| Benzaldehyde | Aromatics | 0.0155 | 0.3648 | 0.6049 | 0.3032 | 0.5529 |
| Methyl phenylacetate | Aromatics | - | 0.1037 | 0.3065 | 0.1036 | 0.5795 |
| P-xylene | Aromatics | 0.0125 | 0.0029 | - | 0.0090 | - |
| toluene | Aromatics | - | - | - | 0.0041 | 0.0077 |
| Phenylacetaldehyde | Aromatics | 0.0115 | 0.2315 | 0.4562 | 0.2755 | 0.6303 |
| 2-methyl-6-ethylaniline | Aromatics | - | 0.0194 | - | - | - |
| Phenylethanol | Aromatics | 0.0958 | 0.5055 | 1.4907 | 0.4552 | 0.7433 |
| N-Ethyl-p-toluidine | Aromatics | - | - | - | - | 0.1267 |
| Benzoic acid | Aromatics | - | - | 0.0078 | - | - |
| Methyl benzoate | Aromatics | - | 0.0042 | 0.1001 | 0.0090 | 0.2632 |
| O-xylene | Aromatics | 0.0108 | - | - | - | - |
| 6-methyl-5-hepten-2-one | Ketones | 0.1297 | 0.0203 | - | 0.0424 | 0.0826 |
| Damanone | Ketones | - | 0.0294 | 0.0268 | 0.0130 | - |
| Anti-3,5-octadien-2-one | Ketones | 0.0309 | 0.0226 | - | 0.0142 | 0.1570 |
| Trans-3-pentene-2-one | Ketones | 0.0068 | - | - | - | - |
| 3,5-octadien-2-one | Ketones | 0.0851 | 0.0221 | - | 0.0384 | 0.1406 |
| 3-hydroxy-2-butanone | Ketones | 0.0810 | - | 0.0049 | 0.0131 | - |
| 2,3-pentanedione | Ketones | 0.0144 | - | - | - | - |
| acetone | Ketones | 0.0206 | 0.0300 | 0.0200 | 0.0306 | 0.1671 |
| 6,10-dimethyl-5,9-undecene 2-one | Ketones | 0.0451 | 0.0172 | 0.0146 | 0.0112 | 0.0989 |
| 2-butanone | Ketones | - | - | - | 0.0049 | 0.0212 |
| Propylene glycol methyl ether | Others | 0.0033 | - | - | - | - |
| 1-Tetradecene | Others | - | - | - | 0.0068 | 0.0918 |
| N-octadecane | Others | - | - | 0.1090 | 0.1149 | - |
| N-heptadecane | Others | 0.0302 | 0.0812 | 0.0599 | 0.0877 | - |
| N. N-dimethylformamide | Others | - | - | 0.0067 | 0.0058 | - |
| Dimethyl sulfide | Others | 0.0103 | 0.0086 | 0.0090 | 0.0088 | - |
| Epoxy propane | Others | 0.0107 | - | - | - | - |
| Ethylsuccinimide | Others | - | - | - | - | 0.0219 |

**Table S2: The contents of volatile flavor compounds in middle part of tobacco leaves (ug/g).**

| **Flavor compounds** | **Group** | **CX10-M-0** | **CX10-M-1** | **CX10-M-2** | **CX10-M-3** | **CX10-M-4** |
| --- | --- | --- | --- | --- | --- | --- |
| N-hexanol | Alcohols | - | 0.0091 | - | - | - |
| Isopentanol | Alcohols | - | 0.0052 | - | - | - |
| Cis-2-pentanol alcohol | Alcohols | 0.0159 | 0.0283 | 0.0577 | 0.2905 | 0.0329 |
| 1-Pentene-3-ol | Alcohols | 0.0052 | 0.0081 | - | 0.4032 | 0.0390 |
| ethanol | Alcohols | 0.0165 | 0.0140 | 0.0309 | 0.3275 | 0.1254 |
| 1-pentanol | Alcohols | - | - | 0.0066 | 0.0837 | 0.0242 |
| Leaf alcohol | Alcohols | 0.0216 | 0.0238 | 0.0867 | - | - |
| styrene | Aromatics | 0.0112 | 0.0139 | 0.0120 | - | - |
| Benzyl alcohol | Aromatics | 0.1105 | 0.5493 | 0.7662 | 0.8055 | 0.1111 |
| Benzaldehyde | Aromatics | 0.2972 | 0.1015 | 0.4161 | 2.1538 | 1.1636 |
| Methyl phenylacetate | Aromatics | - | - | 0.1990 | 1.2599 | 0.4129 |
| P-xylene | Aromatics | - | 0.0114 | - | 0.2343 | - |
| Phenylacetaldehyde | Aromatics | - | 0.0247 | 0.3234 | 2.1912 | 0.5033 |
| 2-methyl-6-ethylaniline | Aromatics | - | - | - | 0.2714 | - |
| Phenylethanol | Aromatics | 0.2943 | 0.3640 | 0.8163 | 3.7686 | 0.5524 |
| N-Ethyl-p-toluidine | Aromatics | - | - | - | - | 0.2048 |
| naphthalene | Aromatics | - | - | 0.0106 | - | - |
| Methyl benzoate | Aromatics | - | - | 0.0345 | - | 0.1603 |
| Acetophenone | Aromatics | 0.0056 | - | - | - | - |
| N-pentanal | Aldehydes | - | - | 0.0207 | 0.5054 | 0.0216 |
| 2-methyl-2-butyraldehyde | Aldehydes | - | - | - | - | 0.0149 |
| Trans crotonaldehyde | Aldehydes | - | - | - | - | 0.0147 |
| Nonanal | Aldehydes | 0.0106 | 0.0540 | 0.2321 | 1.8627 | 0.2827 |
| Tetradecanal | Aldehydes | - | - | 0.0264 | - | - |
| Trans-2-pentenal | Aldehydes | - | - | - | 0.4613 | - |
| Anti-2,4-heptadienal | Aldehydes | - | - | - | 0.5402 | 0.0641 |
| β - cyclic citral | Aldehydes | - | - | 0.0500 | 0.5135 | - |
| Isovaleraldehyde | Aldehydes | 0.0112 | 0.0177 | 0.0914 | 0.1327 | 0.0313 |
| N-hexanal | Aldehydes | - | 0.0177 | 0.0670 | 1.8675 | 0.1721 |
| Trans-2-hexenal | Aldehydes | - | 0.0182 | 0.0373 | 0.5234 | 0.0648 |
| 2-methylacrylaldehyde | Aldehydes | - | 0.0029 | 0.0328 | 0.5821 | 0.1270 |
| 2-methylbutanal | Aldehydes | - | - | 0.0381 | - | - |
| butyrate | Acids | 0.0138 | - | - | - | - |
| Nonanoic acid | Acids | 0.0271 | - | - | - | - |
| 2-methylbutyric acid | Acids | 0.1136 | 0.0424 | - | - | - |
| Isovaleric acid | Acids | 0.0706 | 0.0408 | 0.0365 | - | - |
| 3,3-dimethylacrylic acid | Acids | 0.0236 | - | - | - | - |
| acetic acid | Acids | 0.7535 | 0.5321 | 0.3802 | 1.5369 | 0.2827 |
| Isobutyric acid | Acids | 0.1088 | 0.0410 | - | - | - |
| 6-methyl-5-hepten-2-one | Ketones | 0.0512 | 0.1327 | 0.6360 | 2.8840 | - |
| 6-methyl-3,5-heptadiene-2-one | Ketones | 0.0123 | - | - | - | - |
| 1-Pentene-3-one | Ketones | - | - | - | 0.7219 | - |
| Damanone | Ketones | - | 0.0374 | 0.0250 | 0.2468 | - |
| Anti-3,5-octadien-2-one | Ketones | 0.0679 | - | 0.0673 | 0.3663 | 0.3243 |
| 3,5-octadien-2-one | Ketones | - | 0.0463 | - | 1.1879 | 0.4332 |
| 2,3-Butanedione | Ketones | - | 0.2458 | - | - | - |
| 3-hydroxy-2-butanone | Ketones | 0.0932 | 0.1612 | 0.0813 | 0.6516 | - |
| 2,3-pentanedione | Ketones | - | 0.0094 | - | - | - |
| acetone | Ketones | 0.0374 | 0.0262 | 0.0595 | 0.8234 | 0.2934 |
| 6,10-dimethyl-5,9-undecene 2-one | Ketones | 0.0660 | - | - | - | 0.1497 |
| 2-butanone | Ketones | - | - | - | 0.1448 | 0.0319 |
| Methyl caproate | Esters | 0.0666 | 0.0204 | 0.3203 | 4.0002 | 1.0721 |
| Methyl heptanoate | Esters | - | - | 0.0492 | 0.4792 | 0.0594 |
| Methyl formate | Esters | - | - | 0.0063 | - | 0.0178 |
| Methyl decanoate | Esters | 0.2213 | 0.2581 | - | 17.4718 | 2.9298 |
| Methyl octanoate | Esters | - | - | 0.1429 | 1.0975 | 0.2036 |
| Methyl palmitate | Esters | 0.2776 | 0.2923 | 1.7085 | 12.9504 | 4.3484 |
| Methyl myristate | Esters | - | - | 0.2795 | 1.8951 | 1.0731 |
| Cis-3-hexenoic acid methyl ester | Esters | 0.0775 | 0.0128 | 0.2543 | 0.4676 | 0.0444 |
| Benzyl acetate | Esters | - | 0.0045 | - | - | - |
| Methyl sorbate | Esters | - | - | - | - | 0.0232 |
| Methyl nonanoate | Esters | - | 0.0174 | 0.6573 | 4.7467 | 0.6689 |
| Methyl isobutyrate | Esters | 0.3865 | - | - | - | - |
| Methyl Propionate | Esters | 0.1787 | 0.0086 | 0.0229 | 0.2006 | 0.1340 |
| Butyrate methyl ester | Esters | 0.2813 | - | 0.0106 | 0.1496 | 0.0367 |
| Methyl valerate | Esters | - | - | 0.1733 | 1.1649 | - |
| Hexyl formate | Esters | - | - | 0.0213 | - | 0.0321 |
| Methyl acetate | Esters | 3.7658 | 1.0106 | 0.1772 | 2.0310 | 1.5071 |
| Methyl 2-methylbutyrate | Esters | 0.1178 | - | - | - | - |
| Nicotinic acid methyl ester | Esters | - | - | - | - | 0.0333 |
| Ketamine | Alkaloids | 0.0476 | 0.0775 | 0.0318 | - | 0.3183 |
| Nicotine Diene | Alkaloids | 1.5978 | - | 1.1463 | 9.6670 | - |
| 1'S, 2'S) - Nicotine 1 '- Oxidation | Alkaloids | 0.0440 | 0.0289 | - | - | - |
| nicotine | Alkaloids | 125.0497 | 80.6613 | 81.3477 | 482.9347 | 303.5232 |
| 2,6-dimethylpyridine | Heterocycls | - | - | - | - | 0.0986 |
| 2,6-dimethylpyrazine | Heterocycls | - | - | - | - | 0.0603 |
| pyridine | Heterocycls | 0.0075 | - | - | - | - |
| ligustrazine | Heterocycls | 0.1163 | 0.0978 | - | - | - |
| 3- (4,5-dihydro-1H-pyrrole-2-yl) pyridine | Heterocycls | 0.2537 | 0.1841 | 0.0695 | 0.1517 | 1.2653 |
| 2,5-dimethylpyrazine | Heterocycls | 0.0148 | - | - | - | - |
| 2-ethylfuran | Heterocycls | 0.0175 | 0.0360 | 0.0445 | 0.3774 | 0.0428 |
| 2-n-pentyl furan | Heterocycls | 0.0210 | 0.0800 | 0.1758 | 0.4969 | 0.0768 |
| 2-methylfuran | Heterocycls | - | - | 0.0268 | 0.2559 | 0.0835 |
| 2,3 '- bipyridine | Heterocycls | 0.1185 | 0.0399 | 0.0815 | - | 0.4370 |
| 2,3-dimethylpyrazine | Heterocycls | 0.0136 | 0.0117 | - | - | - |
| Orange blossom alcohol | Terpenoids | - | - | 0.0209 | - | - |
| Plant alcohol | Terpenoids | 0.1382 | 0.3541 | 0.3787 | 4.4520 | 0.8973 |
| Dihydrokiwilactone | Terpenoids | 0.1173 | 0.1338 | 0.3765 | 2.6211 | 0.8418 |
| Geranyl acetone | Terpenoids | - | 0.0573 | 0.1746 | 0.0973 | - |
| β - Violet Ketone | Terpenoids | 0.2517 | 0.2821 | 0.4384 | 2.0109 | 0.6087 |
| 1-Tetradecene | Others | - | - | - | 0.4206 | - |
| N-heptadecane | Others | 0.0360 | - | - | 1.2903 | - |
| N. N-dimethylformamide | Others | - | 0.0164 | - | - | - |
